# Benefits of off-campus education for students in the health sciences: a text-mining analysis

**DOI:** 10.1186/1472-6920-12-84

**Published:** 2012-08-28

**Authors:** Kazumasa Nakagawa, Yasuyoshi Asakawa, Keiko Yamada, Mitsuko Ushikubo, Tohru Yoshida, Haruyasu Yamaguchi

**Affiliations:** 1 Faculty of Health Care, Takasaki University of Health and Welfare, Takasaki City, Gunma, JAPAN; 2 Graduate School of Health Sciences, Gunma University, Maebashi City, Gunma, JAPAN; 3 Comprehensive Regional Support Center, Western Part of Maebashi City, Gunma, JAPAN

**Keywords:** Community-based education, School of health sciences, Early exposure, Role model, Text-mining methods

## Abstract

**Background:**

In Japan, few community-based approaches have been adopted in health-care professional education, and the appropriate content for such approaches has not been clarified. In establishing community-based education for health-care professionals, clarification of its learning effects is required. A community-based educational program was started in 2009 in the health sciences course at Gunma University, and one of the main elements in this program is conducting classes outside school. The purpose of this study was to investigate using text-analysis methods how the off-campus program affects students.

**Methods:**

In all, 116 self-assessment worksheets submitted by students after participating in the off-campus classes were decomposed into words. The extracted words were carefully selected from the perspective of contained meaning or content. With the selected terms, the relations to each word were analyzed by means of cluster analysis.

**Results:**

Cluster analysis was used to select and divide 32 extracted words into four clusters: cluster 1—“actually/direct,” “learn/watch/hear,” “how,” “experience/participation,” “local residents,” “atmosphere in community-based clinical care settings,” “favorable,” “communication/conversation,” and “study”; cluster 2—“work of staff member” and “role”; cluster 3—“interaction/communication,” “understanding,” “feel,” “significant/important/necessity,” and “think”; and cluster 4—“community,” “confusing,” “enjoyable,” “proactive,” “knowledge,” “academic knowledge,” and “class.”

**Conclusions:**

The students who participated in the program achieved different types of learning through the off-campus classes. They also had a positive impression of the community-based experience and interaction with the local residents, which is considered a favorable outcome. Off-campus programs could be a useful educational approach for students in health sciences.

## Background

It is becoming increasingly expected of health-care professionals that they promote not only residents’ health and longevity but also their quality of life. The collapse of the community-based medical system has emerged as a major social problem in Japan, and so the training of health-care professionals involved in community-based medicine is an urgent need. We take it as given that model health-care professionals for future community-based medicine will also be committed to regional development measures.

To meet the social expectations of health-care professionals, it is necessary to implement interdisciplinary and problem-based educational methods to broaden students’ outlooks and enhance their practical abilities in developing a proper professional outlook. O’Toole et al. [1] reported that community-based experience can provide unique, relevant learning opportunities in the context of a professional curriculum, which can complement existing practices in the medical school. Various approaches in community-oriented and community-based medical education have been adopted around the world [2], and it has been reported that a community-oriented medical school curriculum is effective for community-based education [3]. In the field of education for health-care professionals, Davidson et al. [4] introduced a community-based education system based on such core values as disease prevention and teamwork. In Japan, a curriculum that orients students toward community-based medicine has yet to emerge in medical education; however, few approaches have been adopted in allied health-care professional education in this country, and the appropriate content has yet to be determined. To establish community-based education for health-care professionals, clarification of the learning effects is requisite.

In Gunma University, an off-campus education program was initiated as a community-based education program in 2009 as part of the course in health sciences; it received the support of the Ministry of Education, Culture, Sports, Science, and Technology. This off-campus program is part of the university’s health education program for students to gain experience in community-based settings (off-campus classes). It is expected to that this community-based health-care program will facilitate students’ understanding of residents’ needs and activities performed within the community.

The purpose of the present study was to investigate the effect of the off-campus program on students and examine its application.

## Methods

### Overview of off-campus classes

Gunma University has four schools in the Faculty of Health Sciences: Nursing (80 students per grade); Laboratory Sciences (40 students per grade); Physical Therapy (20 students per grade); and Occupational Therapy (20 students per grade). Students’ opportunity to gain experience in community-based settings with off-campus classes is a unique feature of the health education program at this university. The off-campus classes are open to all students, and were designed so that students could participate and acquire experience in project-related activities or events taking place in the local community with attending instructors in charge. For the present study, details relating to project activities or events considered suitable for off-campus learning were collected by university instructors involved in health sciences, and applicants were recruited from among the students.

**Figure 1:**
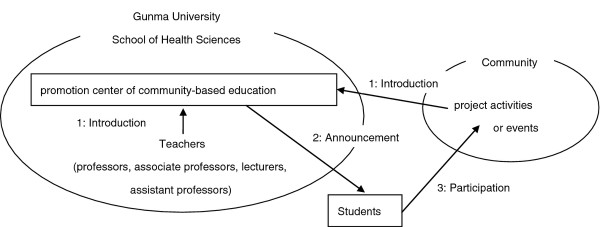
**Outline of the off-campus classes.** 1 (introduction) → 2 (announcement) → 3 (participation).

An outline of the off-campus classes at Gunma University appears in Figure1. To coordinate and manage this program, the Promotion Center of Community-Based Education was established; this involved instructors from each specialty, who supervised overall management and pre- and post-learning of off-campus classes in addition to instructors who taught such classes. The implementation method differed according to the class content, though the number of students (two to five) and duration of each class (3 or 4 hours) were similar among all the classes. Students who participated in this program were required to submit a self-assessment worksheet, summarizing their learning, self-reflection, and general impressions about the class they had participated in, as well as a portfolio with documentation relating to other self-learning outcomes after completing the class.

Since the above program was an optional course, students were free to participate as many times as they wished in their spare time as long as they were students at our university. Students who participated in the off-campus class, which was considered equivalent to an academic credit (approximately 50 hours including pre- and post-studies) in terms of time, were required to submit a report. Academic credits were awarded with the approval of the education committee of our university.

The off-campus classes were roughly categorized into the following six areas of content: (1) contact with local middle-aged and elderly people; (2) contact with disabled people living in the local community; (3) participation in interventions that promoted events conducted in the community; (4) study and experience of activities of health-care professionals working in the community; (5) organizing student-led seminars for local residents; and (6) others. The content of the classes and some examples are presented in Table1. Each class was held just once, and a university lecturer was always in attendance as an instructor.

**Table 1:** Overview and some examples of off-campus classes

**Category**	**Types -times**	**Example**	**Location**	**Number of participants**	**Contents**
Contact with local middle-aged and elderly people	4 types 6 times	Exercise class for middle-aged people	City gym	5	Participation in the class and experience exercises with middle-aged people
		Exercise class for elderly people	Community center	3-5	Experience to provide some exercises for elderly people with staffs
Contact with disabled people living in the local com munity	3 types 5 times	Supporting project for amyotrophic lateral sclerosis (ALS) patients and family members	Public installation	8-10	Participations and experiences in the event supported by the local community for ALS patients and family members
		Supporting project for cancer patients and family members	Public installation	8-10	Participations and experiences in the event supported by the local community for cancer patients and family members
		Group activities with disabled children	Disabled child education instruction	2-3	Participations and experiences in the activities supported by the local community for mentally and physically disabled children and family members
		Group rehabilitation project for persons with higher brain dysfunction and their family members	Community center	3-5	Participations and experiences in the rehabilitation activities for persons with higher brain dysfunction and their family members.
Participation in interventions to promote events conducted in the community	2 types 2 times	Interventions in health promotion and disease prevention events run by the local government	Community hall in the city, etc…	5-8	Participation in events which showing and promoting some interventions or activities for the community-dwelling persons in the community, and experience to run the events with staffs.
Study and experience of activities of healthcare professionals working in the community	3 types 3 times	Study of care managers’ work contents	Care managers’ working places	2-3	Following a care manager and studying her or his work contents in the community.
		Experience of medical checkups for high school baseball players	Practice ground of the high school	3-5	Following a physiotherapist and experiences in some measurements, and studying her or his work contents for sports injury prevention in the community.
		Conducting stretching exercises in the workplace	Working place	3-5	Following a physiotherapist and experiences in some exercise instructions, and studying her or his work contents for working dysfunction prevention in the community.
Organizing student-led seminars for community residents	2 types 3 times	Stretching exercise classes	Public installation	3-5	Experiences to make stretching exercise programs for community-dwelling persons and instruct them.
		Urinary incontinence prevention classes	Public installation	3-5	Experiences to provide information on urinary incontinence prevention for community-dwelling elderly women.
Others	3 types 3 times	Agricultural experience program	Experimental farm	4	Experiences to do farming and think about farmers’ care.
		Interaction with midwives working in the local community	Institutions of midwives	10	Experiences to communicate with midwives working in the local community and think about community-based midwives’ activities.

### Subjects and data collection

After the program was implemented though prior to analysis, informed consent was obtained from participating students. Students were informed that their decision regarding participation would not affect their academic grades and that they were free to withdraw their consent at any time. Students who did not consent to the study were excluded. This study was approved by the Epidemiological Research Ethics Committee of Gunma University Faculty of Medicine (No. 22–13).

In all, 163 students (10 freshmen, 43 sophomores, and 48 junior and 62 senior students; 107 students in 2009, 56 in 2010) participated in the off-campus classes conducted between June 2009 and August 2010. This program consisted of 21 types of class and was conducted 42 times in total. The distribution of the subjects according to their years and specialties is shown in Table2. Among the 163 students, the results from 116, who submitted the self-assessment worksheet, were used for the purposes of this study.

**Table 2:** Number of participants in the off-campus program

**School**	**Nursing sciences**				**Laboratory Sciences**				**Physical Therapy**				**Occupational therapy**				**Total**
**Grade**	**1**	**2**	**3**	**4**	**1**	**2**	**3**	**4**	**1**	**2**	**3**	**4**	**1**	**2**	**3**	**4**	
2009 FY	0	21	17	16	0	0	1	0	4	2	19	21	6	0	0	0	107
2010 FY	0	0	10	15	0	0	0	0	0	3	1	10	0	17	0	0	56
Total	0	21	27	31	0	0	1	0	4	5	20	31	6	17	0	0	163

The off-campus learning experience and general impressions were described in freely written texts in the self-assessment worksheets. The data from these worksheets were converted to anonymous electronic data and used for later analysis. The instructors involved in the students’ education were different from those involved in the data management; therefore, the students’ privacy was protected, and they could not be identified from the electronic data.

### Analytical methods

A text-mining method was used for analysis; this involved using automated procedures for exploiting the enormous amount of knowledge that is available in the biomedical literature [5,6]. In this study, IBM SPSS Text Analytics for Surveys 4 was employed for analysis, which is specialized for mining frequently used individual words; in dealing with a single word, it allows categorization not only by how many times the word is used but how many people use it. All the data were originally in Japanese and analyzed as Japanese words. After the research was completed, all the words were translated into English by the authors for the purposes of this report.

From the list of terms mined, those commonly used by more than 10 students were selected. Various numbers of terms (n = 5, n = 10, n = 15, n = 20, etc.) were tested as cut-off values before we selected the appropriate values; we did this because it was difficult to conduct our analysis using too many occurrences of small numbers of extracted words. We excluded the following terms: those that lacked meaning when used as a single word, such as auxiliary verbs and words specific to the contents of the class; we also excluded those that were not relevant to this study. Terms that referred to the same meaning or content were grouped as single terms. Apart from grouping, all the analytical work was conducted by faculty members, who consulted with one another to ensure whether each word was appropriate for analysis.

Relations among all the remaining terms were analyzed by hierarchical cluster analysis (furthest neighbor method). IBM SPSS 17.0J for Windows was used for the cluster analyses. Analytical results were presented as a dendrogram, within which divisional lines were drawn to determine the lines of understanding; the effect of the program on the students was investigated based on the resulting categories. The appropriate number of clusters was set using Beale’s pseudo F statistic, and the work of drawing lines on the dendrogram was carried out by faculty members, who consulted with one another to ensure which line was the best for appropriate categorizing.

## Results

**Figure 2:**
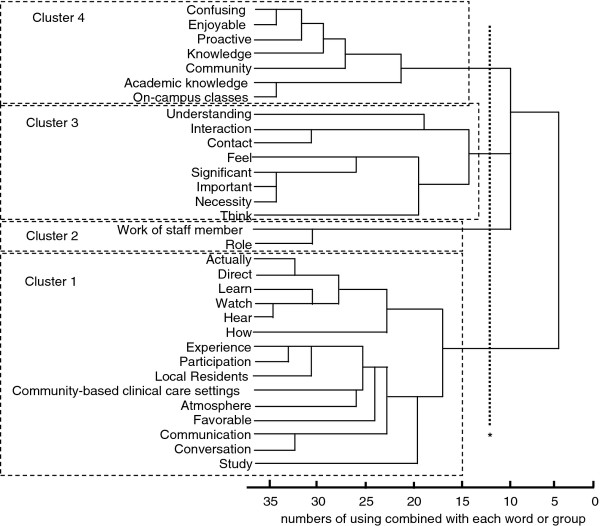
** Relationships among extracted terms (dendrogram).** (* = Line for separation into 4 clusters).

From the 116 self-assessment worksheets, 3,221 terms in 218 categories were extracted by text mining. After we selected the terms commonly observed among more than 10 students, 567 words in 74 categories resulted. Following exclusion and grouping, 32 words were used for the analysis. Cluster analysis produced the following four clusters: cluster 1—“actually/direct,” “learn/watch/hear,” “how,” “experience/participation,” “local residents,” “atmosphere in community-based clinical care settings,” “favorable,” “communication/conversation,” and “study”; cluster 2—“work of staff member” and “role”; cluster 3—“interaction/communication,” “understanding,” “feel,” “significant/important/necessity,” and “think”; cluster 4—“community,” “confusing,” “enjoyable,” “proactive,” “knowledge,” “academic knowledge,” and “class.” The results of the cluster analysis are presented in Figure2.

## Discussion

Japan ranks among the top countries in the world in terms of life expectancy, and as the proportion of its elderly people increases, issues about longevity and way of life for the older population have become increasingly problematic. Further, many health-care professionals have been sent from Japan to work in developing countries. From both a domestic and international perspective, health-care professionals need to develop multi-skilled abilities that are adapted to the residents in their communities. However, few programs for early clinical exposure to community settings have been reported for students in the health sciences (i.e., health-care professional students). At Gunma University, implementation of its off-campus program is the first structured approach in this direction. Analysis of the educational effect of this program may provide with information that will help it include community-based education in the health sciences curriculum.

Regarding cluster 1, students’ overall impression of off-campus classes was considered “favorable.” Since our study results showed a combined use of experience-related terms (“actually/watch/learn/hear”) and terms about the community (“local residents/community-based clinical care settings”), we considered that the off-campus classes were highly evaluated and favorably accepted by the students. We expected the community-based learning to be different from university classroom education, and students who participated in the off-campus classes achieved different types of learning in community settings. This was deemed a favorable outcome since the students obtained a good impression of community intervention. Other reports [7,8] have also noted that community-based medical education programs were rated favorably by students.

Cluster 2, which consisted of “work of staff member” and “role,” was different from the other clusters. It was expected that this cluster would reveal favorable intervention effects, whereby the program would enable students to visualize their futures working in the community by seeing health-care professionals or medical staff actually doing such work. However, the results suggested that university classroom education and the professional roles required in community settings are poorly matched. Community-based education for medical students reportedly motivates the students in understanding the health-care needs of the community and its residents, and at the same time students can learn from health-care professionals working in actual clinical settings [9]; hence, it is expected that the off-campus program at Gunma University will contribute to the education and development of health-care professionals able to work in the community. In contrast, Worley et al. [10] reported on students’ academic performance in tertiary and regional hospitals and found that the effects of community-based clinical practice were no different from those of general practice in hospitals. Their study content, which focused on examination performance by medical students, differs from that in the present study. However, it is expected that an educational approach that enables students to improve their professional knowledge and skills with links to related subjects in other specialty areas will become increasingly adopted in the future.

Regarding cluster 3, the following terms were commonly used: “communication with local residents,” “interaction,” “significant,” “important,” and “necessity.” Martini et al. [11] reported that a community-based curriculum strongly affects students’ career choices. It was also revealed by Howe et al. [12] that physicians’ choosing of careers in community-based settings is closely related to their experience of community-based medical education. Our results suggest that the students became aware of the significance of community interactions and started to become interested in community work. This psychological change is considered an important experience for students during their school years; thus, it is suggested that the students achieved effective learning through the off-campus classes.

Cluster 4 indicated that the students “enjoyed” and were “proactive” in community care settings, though they may have felt some confusion with respect to the actual situation. Some written passages related to these terms—for example, “I didn’t know what I should do, so I was confused” and “I wanted to study in on-campus classes, not to become confused in these settings”—led to the above interpretation. O’Sullivan et al. [13] identified improvement in communication skills and promotion of active learning as relative advantages of community-based clinical education. The present study also found favorable outcomes in that the students enjoyed community-based learning through active participation in the off-campus classes. It is hoped that such off-campus learning experiences will have a synergistic effect on in-campus learning in the future.

Four factors acted as limits in clarifying the effectiveness of off-campus classes in the present study. The first is variety in the content of different classes: every class has its own particular content, and students’ learning may have been influenced by participation in different classes. The second factor is variety among the students: students’ learning and their grades or schools may be closely related, and since community exposure is multi-factorial this alone cannot exert a major effect on the future roles of health-care professionals. The timing of the off-campus exposure may also have an impact on the trainees’ career choices. In this study, we were unable to exclude the influence of such timing, so this needs to be addressed in future studies. Third, only 116 of 163 students submitted the self-assessment worksheets, so it is possible that the results were biased. No attempt was made to obtain information from the 47 students who failed to complete their assessment sheets; future trials would need to collect data from such students. Fourth, no statistical evaluation of the inter-rater agreement was made in this study; every result was judged only by discussion among analyzers. We expected that in this way, we would be able to evaluate every mined term or category statistically to support the results or conclusions.

Seabrook et al. [14] found that a community-based program was beneficial for both community organizations and schools. Members of community organizations were able to obtain information regarding the university curriculum, and schoolteachers had the opportunity to learn about current medical issues and their application in the community. It was reported by Meyer et al. [15] that community-academic partnerships contribute to the overall health of the community, and Calleson et al. [16] demonstrated the importance of the balance between community needs and university requirements for knowledge. The present study was conducted to investigate students’ learning; future studies should investigate outcomes by focusing on the local community, including residents and medical staff members.

## Conclusions

The students who participated in the program achieved different types of learning through the off-campus classes. At the same time, they had a positive impression about community-based experience and interaction with the local residents, which is considered a favorable outcome. Thus, off-campus programs can be a useful educational approach. Along with academic learning, it is necessary to teach health-care professionalism to students within a community context and to examine study outcomes with off-campus programs targeted at a community.

## Competing interests

This study was conducted as a part of a Gunma University educational program, “Practical Health Education Focusing on Quality of Life in the Community—Implementation of Problem-Based Learning Aimed at Human Resource Development for a Society with Health and Longevity,” which was adopted as a good practice program for promoting high-quality university education in 2008 by the Ministry of Education, Culture, Sports, Science, and Technology. All authors read and approved the final manuscript.

## Authors’ contributions

All authors read and approved the final manuscript.

## Pre-publication history

The pre-publication history for this paper can be accessed here:

http://www.biomedcentral.com/1472-6920/12/84/prepub
